# Intraoperative management of Takotsubo cardiomyopathy secondary to parathyroidectomy in the setting of myocardial bridge: a case report

**DOI:** 10.1097/MS9.0000000000004086

**Published:** 2025-10-14

**Authors:** Vladislav Pavlovich Zhitny, Ryan Jannoud, Brett Dixon, Jake Patrick Young, Eugene Tsypin, Ariana R. Carillo, Joelle Sills, Leroy Phillips, Ian Zolnowski

**Affiliations:** a Harvard Medical School, Massachusetts General Hospital, Department of Anesthesia, Critical Care, and Pain Medicine, Boston, Massachusetts, USA; b Department of Anesthesiology, Perioperative Care, and Pain Medicine, New York University, New York City, NY, USA; c Kirk Kerkorian School of Medicine, University of Nevada, Las Vegas, NV, USA; d School of Medicine, Duke University School of Medicine, Durham, NC, USA

**Keywords:** case report, intraoperative arrhythmia, Takotsubo Cardiomyopathy

## Abstract

**Introduction and importance:**

Intraoperative Takotsubo cardiomyopathy (TTC) is a rare, stress-induced condition that mimics acute coronary syndrome and can arise intraoperatively, even during low-risk surgeries. Prompt recognition is essential, as TTC may lead to significant hemodynamic instability in an otherwise stable patient.

**Case presentation:**

We report a 54-year-old female with a history of hyperparathyroidism and prior non-ischemic cardiomyopathy who presented for parathyroidectomy. Despite a negative preoperative cardiac work-up, including stress echocardiogram and transthoracic echocardiogram, the patient developed intraoperative ST depressions and ventricular arrhythmias shortly after thyroid manipulation. Surgical intervention was immediately paused. The patient reverted to normal sinus rhythm, and the surgery was aborted. Postoperatively, troponin levels peaked at 4856. Cardiac catheterization confirmed TTC with apical ballooning and a reduced ejection fraction of 20%. Coronary angiography further revealed a myocardial bridge in the mid-left anterior descending artery with no evidence of obstructive coronary artery disease.

**Clinical discussion:**

The incidental finding of a myocardial bridge offered a critical insight. Dynamic coronary compression during heightened catecholamine stress may have primed the myocardium for collapse. Far from being a benign anomaly, the bridge likely amplified stress-induced dysfunction, reshaping the patient’s risk profile and offering new explanations for prior cardiac symptoms.

**Conclusion:**

This case illustrates how intraoperative TTC can occur unexpectedly, even with a reassuring cardiac history. Careful monitoring, early recognition of arrhythmias, and quick multidisciplinary response are critical to patient safety during surgical procedures.

## Introduction

Takotsubo cardiomyopathy (TTC), also known as stress-induced cardiomyopathy or “broken heart syndrome,” is a transient cardiac syndrome that mimics ST-elevation myocardial infarction (STEMI) in presentation but occurs in the absence of obstructive coronary artery disease. It is named after the Japanese “tako-tsubo,” an octopus trap, due to the left ventricle’s characteristic apical ballooning on imaging^[1]^. The condition is most frequently seen in emotionally distressed, postmenopausal women and is believed to result from a surge in catecholamines that overstimulate the sympathetic nervous system^[2]^.

**HIGHLIGHTS**

- *Uncommon intraoperative presentation of Takotsubo cardiomyopathy*: This case describes the rare onset of Takotsubo cardiomyopathy during an elective parathyroidectomy, despite a normal preoperative cardiac workup and low surgical risk classification.
- *Previously undiagnosed myocardial bridge as a contributing factor*: Postoperative angiography revealed a myocardial bridge in the mid-left anterior descending artery, which likely amplified the catecholamine-induced stress response and contributed to the development of cardiomyopathy.
- *Timely multidisciplinary coordination ensured effective management*: Prompt recognition of intraoperative hemodynamic instability and electrocardiogram changes led to early cardiology involvement, facilitating accurate diagnosis and initiation of appropriate supportive therapy.
- *Highlights a gap in current perioperative risk stratification protocols*: This case underscores the need to consider stress-induced cardiac syndromes like Takotsubo cardiomyopathy in perioperative planning, particularly in patients with underlying structural anomalies not evident on standard testing.

While TTC is often associated with emotional stress, it can also be triggered by physical stressors, including those encountered in the perioperative period. Intraoperative TTC is particularly concerning, as it may present with sudden hemodynamic instability, arrhythmias, or electrocardiogram (ECG) changes, often in patients with no prior cardiac history^[3]^. Diagnosis typically involves echocardiography and cardiac biomarkers to rule out acute coronary syndrome. Management is largely supportive, and while the prognosis is generally favorable, complications such as heart failure or life-threatening arrhythmias can occur^[4]^. Quick recognition and multidisciplinary coordination are essential for creating the best outcomes in affected patients.

TTC typically affects postmenopausal women after acute emotional or physical stress and differs from ischemic cardiomyopathy, which is caused by obstructive atherosclerosis^[5]^. Table 1 outlines the basic distinctions between the two mentioned forms of cardiomyopathy. Unlike a true myocardial infarction, TTC presents with transient left ventricular dysfunction without coronary blockage. We present a rare case of perioperative TTC in the setting of a left anterior descending (LAD) myocardial bridge, underscoring the importance of recognizing TTC in patients with congenital coronary variants. TTC in the perioperative setting is rare, and failure to recognize it promptly can lead to delayed management and adverse outcomes^[3]^. Myocardial bridges, while often asymptomatic, are anatomical variants likely capable of precipitating TTC yet may remain undetected on standard preoperative cardiac evaluation. Their concomitance in this case underscores the need for awareness of such hidden risk factors when acute cardiac events occur intraoperatively. This work has been reported in line with the 2025 Surgical CAse REport (SCARE) criteria^[6]^.

**Table 1:** **Comparing Takotsubo and ischemic cardiomyopathy, emphasizing differences in etiology, clinical presentation, imaging findings, and reversibility of ventricular dysfunction**^[7–10]^

Feature	Takotsubo cardiomyopathy	Ischemic cardiomyopathy
Etiology	Stress-induced (emotional or physical)	Coronary Artery DIsease (CAD); myocardial infarction
Trigger	Sudden stressor (e.g., grief, trauma, illness)	Chronic atherosclerosis or acute coronary occlusion
Patient demographics	Mostly postmenopausal women	More common in men and older adults with CAD risk factors
Symptoms	Chest pain, dyspnea, syncope	Exertional dyspnea, orthopnea, fatigue, chest pain
Coronary angiography	Normal or non-obstructive coronary arteries	Obstructive coronary artery disease is present
LV function/recovery	Good, mostly full recovery	Usually, persistent LV dysfunction
Treatment	Supportive, heart failure therapy	CAD management, revascularization, heart failure therapy

LV, left ventricular.

## Case presentation

### Pre-op

**Figure 1.**
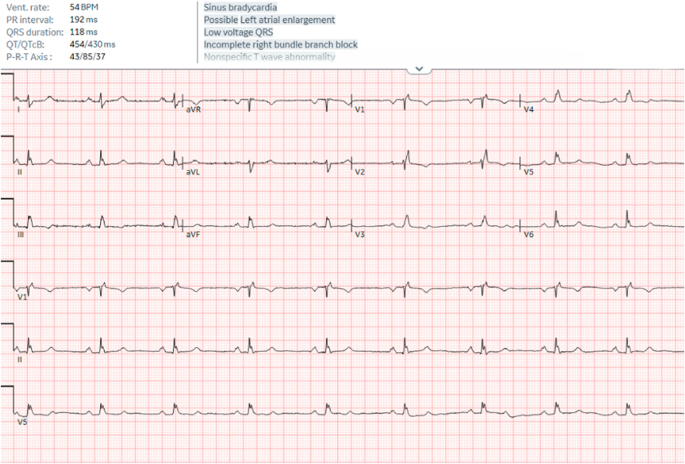
Preoperative ECG.

A 54-year-old female with a complex medical history, including non-ischemic cardiomyopathy, incomplete right bundle branch block (RBBB), unstable angina, asthma, hyperparathyroidism, hypercalcemia, osteoporosis/osteopenia, and stable pectus excavatum, presented for an elective right upper and left lower parathyroidectomy. The patient had undergone an extensive cardiac work-up, including a normal stress echocardiogram several months prior, and a transthoracic echocardiogram (TTE) 1 year prior, which showed a preserved left ventricular ejection fraction (LVEF 53%) with a mild diastolic filling pattern abnormality and no significant valvular disease. A preoperative ECG showed sinus bradycardia, incomplete RBBB, and nonspecific T-wave abnormalities as seen in Figure 1. Findings of stress echocardiogram and TTE were thoroughly reviewed. The final impression was that the patient had no signs of cardiac ischemia, no exercise-induced wall motion abnormalities, and had an average functional capacity for her age.

### Intra-op

The patient was deemed American Society of Anesthesiologists (ASA) Class II and cleared for surgery. General anesthesia was induced with midazolam, fentanyl, lidocaine, and succinylcholine, followed by endotracheal intubation with a 7.0 NIM tube. Anesthesia was maintained with remifentanil and high-dose propofol infusion. Approximately 1 hour into the procedure, during surgical manipulation near the tracheoesophageal groove, the patient developed bradycardia and hypotension, which were initially treated with ephedrine (5 mg) and glycopyrrolate (0.4 mg). Phenylephrine infusion was initiated due to persistent hemodynamic instability.

Soon after, the intraoperative ECG revealed new ST depressions and bigeminy. The surgical team was notified, and the decision to abort surgery and obtain an ECG immediately postoperatively was made. Following cessation of surgical activity, the patient spontaneously reverted to normal sinus rhythm. During emergence, the patient experienced a brief, non-sustained run of ventricular fibrillation lasting less than 10 seconds. Despite this, the patient was extubated successfully and transferred to the post-anesthesia care unit (PACU) on a low-dose norepinephrine infusion.

### Post-op

**Figure 2.**
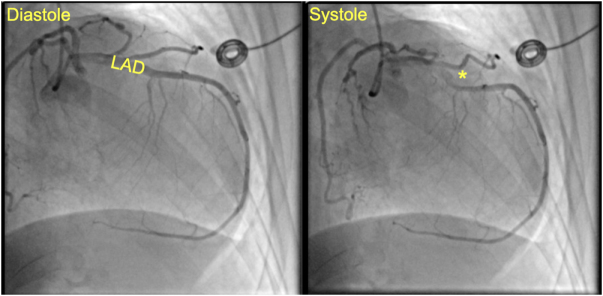
Coronary angiography demonstrating a myocardial bridge limiting systolic perfusion in the mid-left anterior descending (LAD) artery (yellow asterisk), with no evidence of obstructive coronary artery disease.

In the PACU, the patient remained hemodynamically stable without chest pain or shortness of breath. Serial troponins were elevated, peaking at 4856 ng/L. An urgent transthoracic echocardiogram showed a markedly reduced LVEF of 15%, down from the patient’s baseline of 50–53%. Left heart catheterization performed on postoperative day (POD) 1 demonstrated no significant coronary artery disease but did reveal a myocardial bridge in the mid-LAD artery, elevated left ventricular end-diastolic pressure (LVEDP 25 mmHg), and wall motion abnormalities characterized by basal hyperkinesis and mid-to-apical hypokinesis, findings consistent with TTC. LVEF at this time was estimated at 20%. Coronary angiography demonstrating a myocardial bridge in the mid-LAD artery, with no evidence of obstructive coronary artery disease, is shown in Figure 2.

The patient was transferred to the cardiac care unit and weaned off vasopressors by POD 2. On POD 3, guideline-directed medical therapy (GDMT) for heart failure with reduced ejection fraction was initiated, including losartan 12.5 mg daily, selected to reduce afterload, prevent adverse ventricular remodeling, and improve long-term outcomes. Repeat TTE showed modest improvement in LVEF to 25%. On POD 4, spironolactone 25 mg daily and metoprolol tartrate 12.5 mg three times daily were added. Beta-blockade was initiated to reduce myocardial oxygen demand, control arrhythmia risk, and facilitate recovery of ventricular function in stress-induced cardiomyopathy. Prior to discharge, the patient was transitioned to metoprolol succinate 12.5 mg daily, with continuation of losartan (increased to 25 mg daily) and spironolactone. She was also prescribed calcium carbonate 500 mg daily following her parathyroidectomy. Table 2 presents a timeline summarizing the key events.

**Table 2:** Timeline summarizing key events

Phase	Key event
Pre-op	54F for elective parathyroidectomy, normal stress echo, LVEF 53%, ASA II
Intra-op	Bradycardia, hypotension, ST changes, surgery aborted, brief VF, extubated norepinephrine started
Post-op day 0–1	Troponin elevated, LVEF 15–20%
	Cath: no CAD, mid-LAD myocardial bridge
	Takotsubo diagnosis established
Post-op day 2–4	Weaned off pressors
	GDMT started
	LVEF to 25%
Discharge	Stable continued GDMT and calcium carbonate

## Discussion

Intraoperative TTC is a rare but significant event that demands careful monitoring, particularly in patients undergoing even low-risk procedures. Though TTC is more commonly associated with emotional stressors, it is increasingly recognized as a potential consequence of acute physical stress during surgery, including anesthesia induction, surgical manipulation, and autonomic dysregulation^[1]^. Rapidly recognizing TTC and coordinating a treatment plan is highly important, given the condition’s potential for sudden hemodynamic collapse, arrhythmias, and postoperative complications.

Endocrine surgeries, such as parathyroidectomy, are generally classified as low-risk for adverse cardiac events per the 2022 European Society of Cardiology (ESC) guidelines on cardiovascular assessment for noncardiac surgery^[11]^. This patient’s preoperative evaluation incorporated an extensive cardiac work-up, including stress echocardiography and a transthoracic echocardiogram, both of which demonstrated preserved systolic function and no inducible ischemia or significant structural heart disease. Additionally, her functional capacity, as assessed by the Duke Activity Status Index (DASI) and estimated metabolic equivalents (METs > 4), was found adequate to proceed without further noninvasive or invasive testing^[12,13]^.

Despite these reassuring findings, the patient developed acute intraoperative hemodynamic instability with ECG changes and ventricular arrhythmia, later confirmed to be TTC. The perioperative stress response, mediated by elevated circulating catecholamines, inflammation, and sympathovagal imbalance, likely played a key role in precipitating this event. Of note, anesthetic induction agents were selected with careful consideration of the patient’s underlying cardiac conditions, but the physiologic stress of surgical manipulation near the tracheoesophageal groove may have contributed to the acute sympathetic surge that triggered TTC^[14]^.

According to the American Heart Association (AHA) and American College of Cardiology (ACC) perioperative cardiovascular evaluation guidelines, surgery may proceed without delay in patients with stable cardiac conditions, provided they demonstrate adequate functional status and absence of high-risk features such as decompensated heart failure or recent acute coronary syndrome^[15]^. This case underscores a critical limitation of guideline-based risk stratification: even in appropriately evaluated, asymptomatic patients, TTC can manifest unpredictably.

Importantly, this case also revealed a myocardial bridge in the mid-LAD artery on cardiac catheterization, a congenital anomaly that may further complicate left ventricular function by causing dynamic systolic compression of the LAD^[16]^. While often considered benign, myocardial bridges can have functional significance, particularly under conditions of increased demand or elevated catecholamine levels^[16]^. In this patient, the presence of a bridge may have contributed to increased susceptibility of the myocardium to ischemia and stress-induced cardiomyopathy. The intraoperative use of propofol and remifentanil, while typically reducing sympathetic output, was paired with exogenous catecholamines for hemodynamic support. This could have led to an exaggerated adrenergic response. This response could have amplified the hemodynamic consequences of the bridge, contributing to impaired coronary perfusion pressure (CPP), increased ventricular wall stress, and compromised myocardial oxygen delivery. These factors are known to contribute to apical ballooning and hypokinesis observed in TTC. Additionally, the wall motion abnormalities seen in TTC are consistent with stress-induced dysfunction that can be exacerbated by localized ischemia from compression of the LAD^[1]^. Given her prior history of unstable angina, this finding may also provide a pathophysiologic explanation for past symptoms and guide future outpatient management. Recognizing myocardial bridges in the perioperative setting could therefore have important implications for risk stratification and individualized management aimed at preventing TTC and related ischemic complications, including considerations for postoperative care and long-term cardiology follow-up. Surgical unroofing of symptomatic myocardial bridges has been described in the literature and may be considered in select patients with refractory symptoms or recurrent ischemia^[17]^.

## Methods

The patient provided consent for the production of this case report. This case report is devoid of any identifiable protected patient information. The case report is exempt from the Institutional Review Board policy.

## Conclusion

This case illustrates the complexity of perioperative cardiovascular events and the unpredictable nature of TTC, even in patients undergoing low-risk surgeries with otherwise reassuring preoperative evaluations. The patient’s abrupt intraoperative decompensation highlights the importance of maintaining a broad differential diagnosis and the value of rapid interdisciplinary coordination in responding to hemodynamic instability. Additionally, the identification of a myocardial bridge on postoperative angiography adds a layer of diagnostic complexity, offering potential insight into both the acute presentation and the patient’s prior cardiac symptoms. Ultimately, timely recognition of TTC through echocardiography and cardiac catheterization is essential to ensure accurate diagnosis, appropriate management, and long-term follow-up planning.

## Data Availability

Data sharing is not applicable to this article.
